# Effects of Swimming Exercise and L-Carnitine Administration on Insulin Resistance in Juvenile Obese Rats with Type 2 Diabetes

**DOI:** 10.2174/0118715303413143251031170236

**Published:** 2026-01-12

**Authors:** Emrah Yilmaz, Emrah Atay, Ibrahim Kubilay Türkay

**Affiliations:** 1 Department of Coaching Education, Faculty of Sports Sciences, Division of Sports Health Sciences, Süleyman Demirel University, Isparta, Türkiye;; 2 Department of Physical Education and Sports Physical Education and Sports Division, Faculty of Sports Sciences, Mehmet Akif Ersoy University, Burdur, Türkiye;; 3 Department of Sports Management, Faculty of Sports Sciences, Division of Sports Management, Süleyman Demirel University, Isparta, Türkiye

**Keywords:** Regular exercise, diabetes mellitus, l-carnitine, insulin resistance, obesity, swimming exercise

## Abstract

**Introduction:**

Obesity is one of the most common health problems today and is known to cause insulin resistance due to diabetes mellitus (DM). Many treatment methods are still being tested. This study aimed to examine in detail the beneficial effects of exercise (EXE) and L-carnitine (LC) on insulin resistance associated with type 2 DM (DM-2).

**Methods:**

Twenty-four obese male Wistar Albino rats with DM-2 were divided into four groups: DCG, LCG, SEG, and SE+LCG. A 12-week SE and LC administration protocol was applied in the study. Blood serum glucose levels were measured using a blood glucose meter, and insulin levels were determined by an ELISA test. The obtained OD values were used to calculate insulin resistance using the HOMA-IR method. Paired Samples T Test and One-Way ANOVA were used in statistical analyses.

**Results:**

Except for the DCG group, all rats showed significant improvements in body weight, blood glucose levels, and insulin resistance levels. However, no statistically significant differences were found in HOMA-IR values within or between the groups.

**Discussion:**

In disorders observed in juvenile obese rats with Type 2 Diabetes, the combined use of LC and Sbe more effective in enhancing fat burning and facilitating weight loss compared to LC and SE programs applied separately.

**Conclusion:**

LC and SE are effective in reducing obesity, Type 2 Diabetes, and associated insulin resistance levels. Further studies are needed in this field to evaluate the effects of different exercise modalities, treatment types, and durations.

## 1 INTRODUCTION

Today, obesity is almost recognized as a global epidemic. Obesity is defined as excessive amounts of body fat. Overweight and obese individuals are defined by weight and height measurements that provide a person's body mass index, called body mass index (BMI). People with a BMI higher than 30 kg/m2 are considered obese [1]. The primary factors contributing to the development of obesity include the consumption of high-calorie or high-fat foods, insufficient physical activity, and the adoption of a predominantly sedentary lifestyle. Obesity is recognized as a major risk factor for various non-communicable diseases, including cardiovascular disease, DM-2, hypertension, coronary heart disease, and certain types of cancer [2]. Obesity is associated with an increased risk of developing insulin resistance and DM-2 [3]. Insulin resistance is clinically defined as the diminished ability of a specified amount of exogenous or endogenous insulin to stimulate glucose uptake and utilization in an individual, compared to the normal response observed in the general population [4]. Insulin resistance is are strong risk factor for the development of cardiovascular disease and DM-2 [5]. Insulin resistance can be defined as the inability of insulin to stimulate glucose disposal, and when an insulin-resistant person cannot secrete enough insulin to overcome this problem, the person develops DM-2 [6]. Diabetes mellitus (DM) is a multifactorial chronic health condition triggered by many genetic and environmental factors [7-11]. DM is defined as high blood glucose levels due to a deficiency in the concentration and activity of insulin, a pancreatic hormone involved in the control of glycemia. Pharmacologic treatment or insulin may be required to maintain blood glucose levels as close to normal as possible and to delay or possibly prevent the development of DM-related health problems. However, a healthy diet and physical Exercise (EXE) can also help with disease management. To determine the right treatment, identifying the type of diabetes involved plays an important role. DM-2 is often caused by progressive loss of β-cell insulin secretion in the background of insulin resistance [12]. L-Carnitine **(**LC), which has a major role in the conversion of nutrients taken into the body into energy, is a substance that is soluble in water, not easily damaged by heat (200°C), white in color and has a polar structure [13, 14]. One of the important functions of LC, an essential element in the body, is the conversion of fatty acids into energy [15]. LC is also synthesized in the liver and kidneys through methylation of l-lysine and its chemical formula is β-hydroxy-γ-trimethylaminobutyrate [16]. LC has also become a subject of research in different fields of science and studies have shown that the conversion of fatty acids into energy, which is one of the functions of LC in the body, may have positive effects on insulin resistance and DM-2 [17]. Acyl group, propionic acid and toxic compounds can accumulate in the body and cause insulin resistance or heart failure in the heart and skeletal muscle tissues. LC has an important role in preventing these adverse conditions. Accordingly, people with LC deficiency have a high risk of developing cardiovascular diseases and DM [18]. LC and acetyl-LC have been reported to be effective in improving insulin-mediated glucose excretion in both healthy individuals and DM-2 patients [19, 20]. In studies, it was found that serum LC levels decreased in patients with DM-1 and DM-2 [21]. It was found that LC administration had an anti-oxidative effect in rats with STZ-induced DM [22]. However, there is almost no comprehensive study on the effects of these important functions of LC on DM-2 and, consequently on insulin resistance. Although the acute metabolic effects of EXE are mostly due to insulin-independent effects, regular EXE can improve muscle insulin sensitivity and may play an important role in the prevention and treatment of metabolic disorders [23, 24]. Moreover, exercise improves blood lipid profile, fasting blood glucose levels, and insulin sensitivity by increasing daily energy expenditure, which helps regulate metabolic activities and maintain energy balance [25]. The rate of glycogen depletion in EXE is directly proportional to the intensity of EXE. Glycogenolysis is rapid during high-intensity EXE. The process of glycogen breakdown is also regulated by epinephrine levels. High-intensity EXE increases plasma epinephrine levels, which in turn increases glycogen breakdown [26]. It can be said that EXE in DM patients should be performed in order to reduce the need for insulin, increase the transport of glucose required by muscle fibrils, lower plasma blood glucose during EXE and increase glucose uptake into the muscles [27]. While LC and exercise independently improve insulin resistance, their combined effects in juvenile obese rats with DM-2 remain underexplored [28, 29]. This study aims to evaluate the synergistic effects of 12 weeks of swimming exercise and L-carnitine on insulin resistance (HOMA-IR), glucose levels, and body weight in juvenile obese rats with STZ-induced DM-2 [30].

## 2 MATERIALS AND METHODS

### 2.1 Research Model

Combining LC with swimming, a low-impact exercise, may synergistically enhance insulin sensitivity in juvenile obese rats, a model relevant to early-onset DM-2 [31, 32].

### 2.2 Ethical Approval for Animal Studies

All experiments were performed in accordance with ARRIVE (Animal Research: Reporting *in vivo* Experiments) guideline 2.0. The experimental study protocol was approved by Burdur Mehmet Akif Ersoy University Animal Experiments Local Ethics Committee (MAKÜ-HADYEK) (Ethics No: 18.03.2020/631). Turkey. Twenty-four male Wistar Albino rats, aged 3-6 months, were obtained from the Burdur Mehmet Akif Ersoy University Experimental Animal Production and Research Center. In order to prevent metabolic changes caused by gender differences from affecting the results of the study, only male Wistar albino rats were used in the study. The rats were housed in Rital brand polycarbonate laboratory rodent cages under controlled conditions of constant temperature (22±2ºC) and humidity (55-60%) for 12 hours of light and 12 hours of darkness. Each cage contained six rats from the same group.

### 2.3 Feeding Obese Rats

Rats weighing at least 300 g were included in all groups in the study and fed with standard pellet rat feed for twelve weeks. The feed compartments of the cages where the rats were housed were checked at regular intervals, and the missing feed was replenished to ensure that the feed compartments were not empty.

### 2.4 Induction of DM-2

 Cayman brand Streptozocin (STZ) was used to induce DM-2 in rats. STZ was administered intraperitoneally (i.p.) at a dose of 35 mg/kg [33] to rats with obesity (body weight >300 g) to induce a DM-2 model. This dose was given to each animal on the first day of the study. Two days after the administration of STZ, only 20 out of 30 rats, whose tail vein blood glucose levels exceeded 200 mg/dl, were included in the study. STZ was re-administered to the remaining 10 rats. Two days later, 4 more rats whose tail vein blood glucose level was higher than 200 mg/dl were included in the study, and the experiment was started. The reason why six rats could not be included in the study was that DM-2 could not be produced in rats.

### 2.5 Study Groups

 Twenty-four male Wistar Albino rats aged 3–6 months with type 2 diabetes mellitus (DM-2) were randomly divided into the following four groups: 1-Diabetic Control Group (DCG) (n=6): No treatment other than standard nutrition was administered to the DM-2 induced DCG. 2-L-Carnitine Group (LCG) (n=6): LC administration by gavage at a dose of 300 mg/kg [33] 3 days a week with standard nutrition. 3-Swimming Exercise Group (SEG) (n=6): Regular swimming EXE with standard nutrition. 4- Swimming Exercise+L-Carnitine Group (SE+LCG) (n=6): LC gavage method before EXE 3 days a week with standard nutrition and regular swimming EXE afterwards. Before starting the experiment, a total of 24 rats were randomly assigned to study groups with 6 rats in each group. At the beginning of the first week, as well as at the end of the fourth, eighth, and twelfth weeks of the experiment, the rats' weight and blood glucose levels, obtained from the tail vein, were measured.

### 2.6 Exercise Protocol

Before starting the study, the pool water was adjusted to a level where the rats' noses remained outside when their hind feet touched the ground to prevent stress in the rats. In addition, swimming training was provided to all rats before the study began, ensuring that they reached a level where they could swim in the pool water without stress. In the study, the experimental groups, SE+LCG and SEG, were given swimming exercise regularly for 30 minutes, 3 days a week. The exercise period lasted for a total of 12 weeks (3 months), excluding the period during which the rats underwent swimming training. Efforts were made to ensure that the rats were not stressed or exhausted during the exercise. The water temperature was scientifically maintained at 23°C, and a thermostat was used to prevent any harm to the rats.

### 2.7 L-carnitine Administration

Only LCG and SE+LCG were administered LC three times a week at a dose (300 mg/kg) [34] determined to be appropriate for rats at the daily amount administered to humans in liquid form orally just before starting EXE. On the 1st day of the study, at the end of the 4^th^ week and at the end of the 8^th^ week, the weight of the rats was measured, and the amount of LC (300 mg/kg) administered to the rats was calculated according to the weight changes.

### 2.8 Blood Sampling from Rats

In accordance with animal ethics guidelines, approximately 30-40 µl of blood was collected from the tail vein of rats with full stomachs into Eppendorf tubes containing an appropriate anticoagulant solution. Blood samples were centrifuged using an Eppendorf 5415 R Eppendorf (Germany) device, and plasma was separated. Blood was collected from the rats two days after the first and last EXE day of the study. The main purpose of taking blood two days later is to ensure the regeneration and recovery of cells and tissues that are destroyed and fatigued immediately after swimming EXE. In this way, a more efficient and reliable result was obtained.

### 2.9 Biochemical Analysis

Blood samples taken from the rats at the beginning and end of the study were examined for biochemical analyses at the Animal Hospital Laboratory of the Faculty of Veterinary Medicine, Mehmet Akif Ersoy University, Burdur, Turkey.

### 2.10 Measurement of Serum Glucose Levels

The glucose content in sera was analyzed spectrophotometrically using Randox chemical kits and a Randox Monaco RX5002 analyzer (Randox Monaco, UK).

### 2.11 Measurement of Serum Insulin Levels

Rat Insulin ELISA Kit (Cat.No E0707Ra, BT LAB, China) was used for quantitative measurement of venous blood insulin level. The “double antibody sandwich” method was used for ELISA. When the test was performed, the steps in the procedure were followed completely. 1- All reagents, standard solutions, and samples were prepared following the instructions provided. Before use, all reagents were brought to room temperature. The procedures were carried out at room temperature. 2-The number of wells required for the number of samples was determined. Wells were placed in frames. 3-A volume of 50 µl of standard solution was added to the standard well. No antibody was added to the standard well, as the standard solution already contains the antibody. 4-A volume of 40 µl of sample was added to each sample well, followed by 10 µl of antibody. Next, 50 µl of streptavidin-HRP was added to both the sample and standard wells, and the contents were thoroughly mixed. The plate, containing both samples and standards, was then covered and incubated in the dark at 37°C for sixty minutes. 5- After the incubation period, the cover slip was removed, and the plate was washed five times with wash buffer. Each well was rinsed with at least 0.35 ml of wash buffer, allowing thirty seconds to one minute per wash. The plate was then dried quickly by inverting it onto a paper towel and shaking it. 6-Each well received 50 µl of substrate solution A, followed by 50 µl of substrate solution B. The plate was then covered with a fresh cover slip and incubated in the dark at 37°C for ten minutes. 7- Next, 50 µl of stop solution was added to each well, causing the blue color to change to yellow. 8- The optical density (OD) of each well was measured at 450 nm using a microplate reader within ten minutes of adding the stop solution.

### 2.12 Calculation of Insulin Resistance

 The obtained OD values were used to calculate insulin resistance using the HOMA-IR method. HOMA-IR values were calculated with the formula: HOMA-IR = (fasting serum insulin level (μU/mL) x fasting serum glucose level (mmol/L)) / 22.5 [35, 36].

### 2.13 Statistical Analysis

Statistical analysis for the study was conducted using the IBM SPSS Statistics software, version 22.0 (SPSS Inc., Chicago, IL, USA). The Shapiro-Wilk test, which is commonly preferred for small data sets, was used to assess the normality of all values. The test results showed that all values were normally distributed. A one-way ANOVA test was used to compare the time-dependent weight changes of the groups in independent groups (DCG, LCG, SEG, SE+LCG) between weeks. Tukey HSD Post Hoc test was used for comparisons of time-dependent weight changes of each group. A one-way ANOVA test was used for intergroup comparisons of time-dependent weekly weight changes. Paired Samples T Test was used to compare the pre-test and post-test glucose values of each group. A one-way ANOVA test was used in the comparison of pre-test and post-test glucose values between groups. Paired Samples T Test was used to compare the pre-test and post-test insulin resistance values of each group. One-Way ANOVA test was used in the comparison of pre-test and post-test insulin resistance values between groups. All test results were evaluated with a margin of error of 0.05.

## 3 RESULTS

**Fig. (1):**
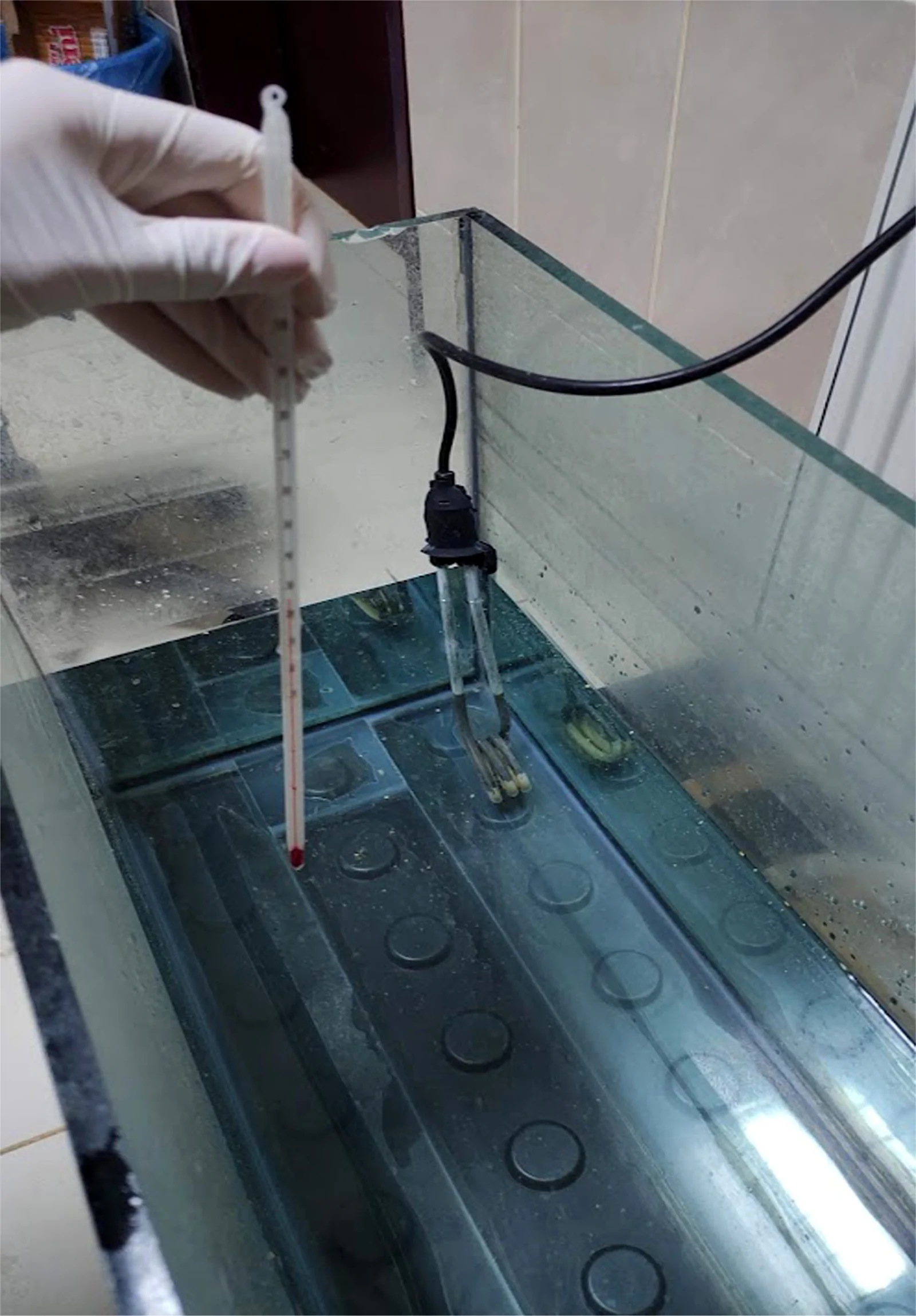
Pool realising swimming EXEs.

The water temperature of the pool where the swimming exercises were performed was scientifically maintained at 23°C, and a thermostat was used to prevent any harm (Fig. **1**).

**Fig. (2):**
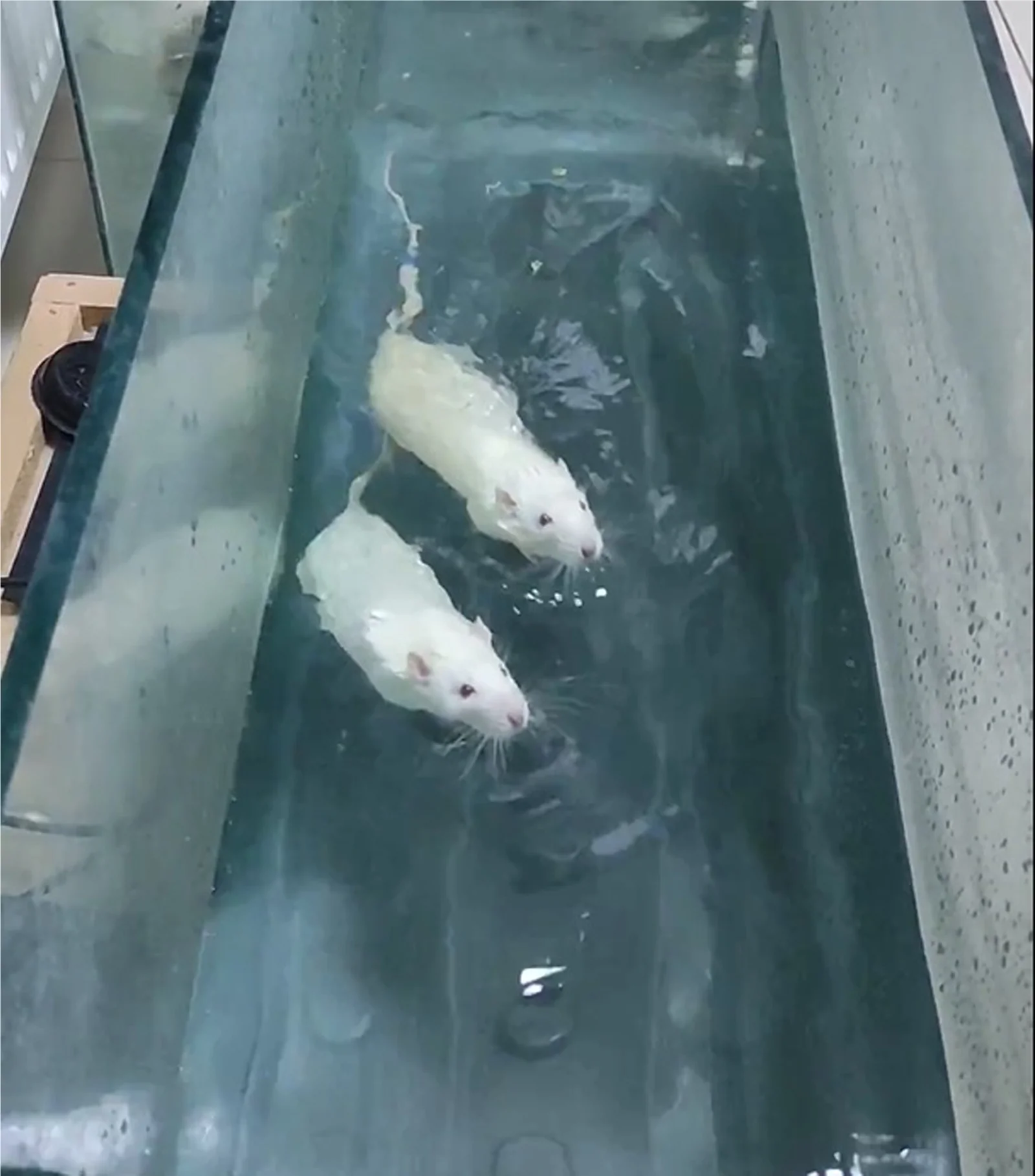
The rats performing swimming EXEs.

To prevent rats from experiencing stress during swimming training, the pool water was adjusted so that their noses remained above water when their hind legs touched the bottom (Fig. **2**).

### 3.1 12-week findings related to weight variables

When the mean weight values of all groups between weeks were analyzed, DCG rats gained weight regularly every week. LCG rats lost weight from week 1 to week 4, and then their weight increased regularly from week 8 to week 12. The weight of SEG rats remained constant from week 1 to week 4 and increased at week 8 and week 12. The weight of SE+LCG rats increased from week 1 to week 4, but weights decreased steadily at week 8 and week 12.

When the weight values of the groups were compared between weeks, the difference was found to be statistically insignificant (*p*˃ 0.05) (Table **1**).

 There was no statistical difference (*p*˃ 0.05) in the weight values of DCG rats between weeks (Table **2**).

 There was no statistical difference (*p*˃ 0.05) in the weight values of LCG rats between weeks (Table **3**).

 There was no statistically significant difference (*p*˃ 0.05) in the weight values of SEG rats between weeks (Table **4**).

There was no statistical difference (*p*˃ 0.05) in the weight values of the rats in the SE+LCG group between the weeks (Table **5**).

When the weight values of the rats in all groups were compared between the groups according to weeks, the differences were found to be statistically insignificant (*p*˃ 0.05) (Table **6**).

### 3.2 Findings Related to Glucose Variable

When the pre-test and post-test averages of glucose values of all groups were examined, an increase was found in the pre-test and post-test glucose values of CG rats, while a decrease was found in the pre-test and post-test glucose values of LCG, SEG and SE+LCG rats.

There was no statistical difference (*p*˃ 0.05) in the pre- and post-test glucose values of the groups (Table **7**).

When the pre- and post-test glucose values of all groups were compared between the groups, the difference was found to be statistically insignificant (*p*˃ 0.05) (Table **8**).

### 3.3 Findings Related to the Insulin Resistance Variable

When the pre- and post-test averages of insulin resistance values of all groups were examined, a decrease was found in the pre- and post-test insulin resistance values of the rats in all groups.

There was no statistically significant difference (*p*˃ 0.05) between the pre- and post-test insulin resistance values of the groups (Table **9**).

When the pre- and post-test insulin resistance values of all groups were compared between the groups, the difference was found to be statistically insignificant (*p*˃ 0.05) (Table **10**).

## 4 DISCUSSION

In obesity-related DM-2 and related insulin resistance disorders, regular EXE before the disease is a preventive measure in the development of the disease [37-40], and regular EXE in people with obesity and type 2 diabetes is of great importance in the course and treatment of the disease [41-43]. In the study, it was determined that DCG rats gained weight regularly every week from the first week to the twelfth week in juvenile obese rats with DM-2. Since the rats were obese and diabetic at the beginning of the study, it is possible that their insulin sensitivity was impaired, their body fat tissue ratio was high with the current BMI, and their metabolic rate slowed down. At the same time, the weight of the rats will inevitably increase regularly due to the continuation of their routine eating habits for twelve weeks and their inactivity. In a study investigating the anthropometric parameters and markers of male adult Wistar rats, it was reported that obese rats continued to gain weight regularly with age and as a result of a sedentary environment [44]. LC is a molecule of great importance in the body, synthesized in the liver and kidneys. In addition to its role in energy production, it is responsible for transporting long-chain fatty acids across the mitochondrial membrane and detoxifying organic acids [45]. The external intake of LC increases fat burning in the body, preventing the accumulation of excess fat tissue, and regulates insulin resistance associated with obesity and DM-2 [46-48]. In the study, it was observed that LCG rats lost weight from the first week to the fourth week, and their weight gradually increased from the fourth week to the twelfth week, but their weight at the twelfth week was still lower than their weight in the first week. According to the study findings, during the first four weeks of LC use, it was observed that LC activated existing fat tissues and accelerated energy utilization, leading to weight loss. In the following four-week period, it was noted that the rats using LC did not lose weight; on the contrary, they gained weight. This suggests that LC may initially stimulate energy metabolism rapidly, leading to weight loss, and then reduce fat tissue while increasing muscle mass. In the last four weeks, the continued weight loss can be attributed to the increased muscle tissue and reduced fat tissue, which may have reactivated the previously stagnant weight loss due to an accelerated metabolism. Salmanoğlu *et al.* found that, in the fifth week of the study, the rats' weight was higher than their initial weight; however, at the end of the study, it was determined that the rats' current weight was lower than their weight at the beginning of the study [49]. In another study investigating the effect of LC on weight, it was reported that LC use affected weight gain in obesity and led to a tendency for less weight gain [50]. In the study, it was found that the weight of the SEG rats remained stable from the first week to the fourth week, but increased after the fourth week, continuing through the eighth and twelfth weeks. It is an undeniable fact that regular EXE induces muscle hypertrophy. According to the study's findings, rats that performed regular EXE for twelve weeks experienced weight gain. This is thought to be an acute increase related to EXE-induced muscle hypertrophy. Zhang and colleagues applied both acute and chronic swimming EXEs to obese rats, finding that the weight of the rats subjected to acute EXE increased in the ninth week, similar to the EXE group in our study, while the group subjected to chronic swimming EXE showed a decrease in weight by the seventeenth week [51]. In the study, it was observed that the weight of the SE+LCG rats increased from the first week to the fourth week but then decreased consistently from the fourth week through the eighth and twelfth weeks. Considering the findings of the SEG and LCG groups separately, it is believed that the initial weight gain during the first four weeks resulted from muscle hypertrophy due to regular EXE. However, with the introduction of LC, the continued weight loss is thought to be due to an increase in metabolic rate and muscle mass. The separate findings from these two groups clearly support the results of the EEX+LCG group. Similar findings were also observed in a study involving rats fed a high-fat diet and a normal diet [52]. Additionally, the use of LC in diabetic rats was found to significantly reduce triglyceride levels, and it was determined that LC could also lower cholesterol levels, suggesting its potential therapeutic use [53]. In the study, no statistically significant differences were found in weight changes between the weeks (1^st^, 4^th^, 8^th^, and 12^th^ weeks) across the four groups (DCG, LCG, SEG, SE+LCG). Although weight fluctuations, both gains and losses, were observed between the weeks within the groups, it is believed that these changes were statistically insignificant due to the short duration of EXE and LC use, limiting their effects on metabolism. In the study, when the weight measurements of the groups were compared by weeks, no statistically significant differences were found. Although the differences in weight measurements across the weeks were not statistically significant, it was observed that the LCG group had lower average weights compared to the DCG group, which continued its routine diet and led a sedentary lifestyle. In the first week, DCG rats were found to weigh less than LCG and SEG rats, but after the fourth week, LCG experienced rapid weight loss, and by the eighth and twelfth weeks, LCG rats had lower weights than DCG rats. The situation for the SE+LCG group was somewhat different from the other groups. Compared to the DCG group, the SE+LCG group had higher average weights in the first, fourth, and eighth weeks, but by the twelfth week, their average weight dropped rapidly, becoming lower than that of the DCG group. According to these findings, EXE and LC use are effective in protecting against and treating obesity and other diseases that may be triggered by obesity, as also noted in the literature [54-58]. In the study, it was observed that the glucose levels of the DCG rats increased over the following weeks. This can be attributed to hormonal imbalances related to DM-2 in the rats. Özköslü and colleagues applied different EXE models to rats with DM-2 and found that the glucose levels of the rats in the DCG, which did not undergo EXE and continued their routine daily diet, showed a consistent increase during the eight-week study period [59]. In the study, a decrease in the glucose levels of the LCG group was observed. This can be attributed to LC facilitating and increasing the entry of glucose into cells, thus converting glucose in the body into energy, leading to a reduction in glucose levels. Salmanoğlu and colleagues found that the fasting glucose levels of DM-2 rats treated with LC showed a significant decrease by the end of the study. Wang and colleagues noted that in order for LC to be effective on fasting plasma glucose levels, it should be taken in a dosage of two grams per day for at least 36 weeks [60]. In the study, a decrease in glucose levels was observed in the SEG rats, though not as significant as in the LCG rats. These findings suggest that regular exercise reduces glucose levels in the body. Therefore, it is believed that regular EXE could lower glucose levels in individuals with DM-2. The findings of Özköslü and colleagues, which are similar to the glucose levels of the DCG group in our study, also show a resemblance to the glucose levels of the SEG. In their study, the combined SEG showed a regular decrease in glucose levels at the second, fifth, and eighth weeks. In the study, a more significant reduction in glucose levels was observed in the SE+LCG rats compared to the LCG and SEG rats. Considering the glucose level decreases observed in the previous two groups, it was determined that the combined use of EXE and LC led to a more effective reduction in glucose levels. Therefore, it is believed that regular EXE supports the use of LC, and LC, in turn, enhances the effects of regular EXE, thus supporting the treatment of DM-2. Snowling and Hopkins noted that any type of EXE performed over twelve weeks in DM-2 patients provided benefits similar to those of medication, and that combined EXEs were more effective in glucose control than aerobic and resistance EXEs alone [61]. Numerous studies in the literature highlight the importance of EXE in regulating blood glucose levels in DM-2 patients [62-67]. Hadadinezhad and colleagues found that LC significantly reduced fasting blood sugar levels in twelve weeks but had no effect on postprandial blood sugar levels or HbA1c concentration values in non-insulin-dependent DM patients [68]. In the study, it was found that the highest glucose levels were in the DCG rats, followed by the LCG, then the SEG, with the lowest glucose levels in the SE+LCG for both the pre-test and post-test values. Although the differences were statistically insignificant, when the pre-test and post-test glucose values of the groups were examined separately, it was observed that the post-test glucose values of the DCG were higher than their pre-test values. In contrast, the post-test glucose values of the other groups were lower than their pre-test values. This indicates that exercising, using LC, and applying both methods benefited the management of fasting glucose levels. Unlike the aerobic swimming EXEs we implemented, Agustini and Dewi conducted resistance EXEs with rubber bands in their studies, finding that resistance EXEs reduced blood glucose levels in DM-2 patients [69]. Based on these results, we can say that different types of EXE methods [70] applied to individuals with DM-2 have positive effects on patients' blood glucose levels. In our study, SEG and SE+LCG EXEs were performed in the afternoon. Savikj and colleagues investigated the effects of EXE on blood glucose levels based on the time of day in DM-2 patients and applied HIIT EXE methods in two different time frames: morning and afternoon. The study found that afternoon HIIT exercises were more effective than morning sessions in improving blood sugar levels. Another significant finding was that morning HIIT EXEs had an acute adverse effect, increasing blood glucose levels [71]. Karalis *et al;* conducted a study involving two groups, one with DM-2 and one without, where both groups were given two grams of LC orally once a day for six months. After three months, acute improvements in glucose levels were observed in the patients, but no chronic effects of the treatment were noted [72]. There has been no literature on the combined treatment of EXE and LC in DM-2 patients. It is thought that this aspect of the study findings will contribute to the literature. In the study, a decrease in insulin resistance values was observed in all groups of rats. Krishna and colleagues explained that the reduction in insulin resistance values across all groups could be attributed to the induction of diabetes through STZ, which may result in decreased serum insulin levels in the rats [73]. In the study, a slight decrease in insulin resistance values was observed in the LCG. Ranjbar Kohan *et al;* reported that the insulin resistance values of the DCG rats with DM-2 increased until the fourteenth day and showed a decrease by the twenty-eighth day compared to the first day of the study [74]. In contrast, the insulin resistance values of the rats in the LCG decreased steadily throughout the study, showing a significant reduction by the twenty-eighth day compared to the initial insulin resistance values. The findings of this study support the decrease in insulin resistance values in the DCG and LCG groups of rats in our study. In another study in the literature, sibutramine, a medication used for weight loss, weight maintenance, and obesity treatment, was combined with LC and administered to DM-2 patients, resulting in a significant improvement in the patients' insulin resistance values [75]. In the study, similar to the decrease in insulin resistance values in SEG rats, George and colleagues applied aerobic EXEs to overweight obese girls and found improvements and enhancements in the girls' insulin resistance values compared to their initial insulin resistance values [76]. In a different study involving DM-2 rats, aerobic swimming EXE and melatonin were administered, and it was thought that the group receiving melatonin along with EXE showed significant decreases in insulin resistance values, thereby increasing EXE performance and potentially reducing the risk of hypertension associated with DM [77]. Özköslü investigated the effects of aerobic, resistance, and combined training on insulin resistance in DM-2 rats and found that the group with the highest insulin resistance was the control group that did not EXE, while the group with the lowest insulin resistance was the one that engaged in aerobic EXE. Current studies in the literature also indicate that L-carnitine increases β-oxidation and enhances exercise insulin signaling [78, 79]. However, there were no statistically significant differences in insulin resistance values between the groups [80]. In the study, combined SE and LC modestly reduced insulin resistance in juvenile obese rats with DM-2, but statistical significance was not achieved. In the study, similar to the insulin resistance values in SE+LCG rats, Hamasaki found that interval training affected insulin sensitivity in DM-2 patients and had therapeutic implications [81]. Cresto *et al;* observed improvements in the insulin resistance values of DM-2 rats treated with LC, and by the end of the study, these values had decreased to levels comparable to those of healthy normal-weight rats [82]. Molfino *et al;* determined that the administration of oral LC improved insulin resistance in DM-2 patients [83]. In the study, it was determined that at the beginning and end of the intervention, the highest insulin resistance value in DM-2 rats was in the LCG, while the lowest insulin resistance value was in the SE+LCG; however, the differences in these insulin resistance values between the groups were not found to be statistically significant. El-Sheikh *et al;* found that the use of glimepiride, an anti-diabetic medication, in conjunction with LC had positive effects on insulin resistance values, and they even stated that the use of LC alone was more beneficial than glimepiride in terms of direct and indirect biomarkers of insulin resistance [84]. Boghrabadi *et al;* determined that twelve weeks of aerobic treadmill EXE, three times a week at an intensity of 60 to 70%, for 40 minutes, was therapeutically effective in terms of fasting blood glucose and insulin resistance values in DM-2 patients [85]. As seen in studies in the literature on this topic, both EXE and LC have been indicated to be important in the treatment of DM-2; however, the findings of this study are thought to contribute to the literature by revealing how the combination of these two interventions can have a therapeutic effect. To provide healthier data, it is suggested that the effects of EXE, LC, or both on the insulin resistance of rats should be examined more frequently in 4-week periods. The American College of Sports Medicine (ACSM) and the American Diabetes Association (ADA), in their joint statement published simultaneously in Medicine & Science in Sports & Exercise and Diabetes Care, which was written and approved by the ADA Board of Directors in July 2010, clearly stated that EXE plays an important role in the prevention and management of insulin resistance, DM-2, and health complications related to DM. They noted that both aerobic and resistance training can improve insulin sensitivity, at least in the short term, and may help regulate quality of life; however, to maintain these benefits, EXE needs to be performed regularly. They emphasized that many individuals with DM-2 can safely engage in exercise as long as certain precautions are taken, and that diversifying and increasing physical activity or EXE program is critical for optimal health in individuals with DM-2 [86].

## CONCLUSION

In conclusion, excessive energy intake combined with a sedentary lifestyle may contribute to the persistence of the current condition in DM2 patients. This lifestyle, characterized by low levels of physical activity and inadequate dietary habits, may increase metabolic imbalances and promote insulin resistance. A 12-week period of regular EXE in these patients can help reduce insulin resistance and glucose levels. This improvement may occur due to increased glucose uptake by skeletal muscles, enhanced insulin sensitivity, and regulation of adipose tissue function. Additionally, LC may help reduce insulin resistance and glucose levels in DM-2 patients even in the absence of EXE. This is because LC facilitates the transport of long-chain fatty acids into mitochondria, supporting the beta-oxidation process, which in turn improves energy metabolism and indirectly enhances glucose regulation by reducing lipid accumulation. Finally, the combination of regular EXE and LC supplementation may be effective in alleviating the negative aspects of DM-2. This synergistic effect is thought to result from their complementary actions on cellular metabolism and lipid utilization. However, special attention should be given to the first four weeks of LC use in combination with regular EXE. This period is critically important due to the simultaneous occurrence of physiological stress caused by the new exercise regimen and the metabolic changes associated with LC supplementation. In the initial phase of adaptation to regular EXE, hormonal changes occur, which may affect energy balance and glucose metabolism. Subsequently, LC enhances mitochondrial function within cells and activates fat cells. This process supports the utilization of stored fat as an energy source and may improve overall metabolic health. It may lead to a temporary plateau in weight loss; however, in the long term, it can be effective in achieving targeted weight loss and reducing insulin resistance and glucose levels. Therefore, it is recommended that DM-2 patients use LC in conjunction with regular EXE for a period of 12 weeks. This duration may not always be sufficient to provide significant physiological adaptations and clinical improvements, as individual responses can vary depending on baseline levels and adherence to the intervention. In addition to the findings of this study, future research may involve longer durations, different types of EXE, and applications of increased workloads at various intensities. For instance, resistance training, interval training, or aquatic exercise could be explored for their comparative or combined effects. It is also recommended that insulin resistance parameters be examined in detail at four-week intervals. Such time-based analyses would allow researchers to detect early, mid-term, and late responses to the intervention and offer more comprehensive insights into the course of metabolic changes. In addition to the glucose and insulin resistance parameters analyzed in this study, other indicators such as inflammatory markers, oxidative stress indicators, mitochondrial biogenesis factors, lipid profiles, and diverse body composition metrics could also be evaluated. The regular exercise and L-carnitine protocol used in our study may potentially be adapted to other disease types, and their effects could be investigated accordingly. Chronic conditions such as obesity, metabolic syndrome, cardiovascular diseases, and even neurodegenerative disorders may benefit from such interventions, making this field of research highly significant for broader clinical applications.

## Figures and Tables

**Table 1:** Statistical analysis results of the weight values of groups between weeks.

Groups	Week	Mean (gram)	SD	F	*p*-value
DCG	Week 1	392.00	41.47	0.467	0.708
	Week 4	417.58	55.05		
	Week 8	424.33	60.71		
	Week 12	427.41	69.66		
LCG	Week 1	406.00	66.14	0.399	0.755
	Week 4	372.00	36.59		
	Week 8	391.00	57.51		
	Week 12	402.41	71.14		
SEG	Week 1	407.66	52.86	0.093	0.963
	Week 4	407.16	66.10		
	Week 8	418.16	66.70		
	Week 12	423.41	70.33		
SE+LCG	Week 1	414.83	72.61	0.205	0.892
	Week 4	447.08	98.24		
	Week 8	445.16	98.30		
	Week 12	418.75	96.86		

**Abbreviations:** DCG: diabetic control group, LCG: l-carnitine group, SEG: swimming exercise group, SE+LCG: swimming exercise+l-carnitine group, SD: standard deviation. One-way ANOVA.

**Table 2:** Findings of DCG weight values between weeks.

(I) Week	(J) Week	Mean Difference (I-J)	*p*-value
Week 1	Week 4	-25.58	0.868
	Week 8	-32.33	0.767
	Week 12	-35.41	0.715
Week 4	Week 1	25.58	0.868
	Week 8	-6.75	0.997
	Week 12	-9.83	0.991
Week 8	Week 1	32.33	0.767
	Week 4	6.75	0.997
	Week 12	-3.08	1.000
Week 12	Week 1	35.41	0.715
	Week 4	9.83	0.991
	Week 8	3.08	1.000

**Note:** One-way ANOVA, Tukey HSD Post Hoc.

**Table 3:** Findings of LCG weight values between weeks.

(I) Week	(J) Week	Mean Difference (I-J)	*p*-value
Week 1	Week 4	34.00	0.755
	Week 8	15.00	0.971
	Week 12	3.58	1.000
Week 4	Week 1	-34.00	0.755
	Week 8	-19.00	0.944
	Week 12	-30.41	0.811
Week 8	Week 1	-15.00	0.971
	Week 4	19.00	0.944
	Week 12	-11.41	0.987
Week 12	Week 1	-3.58	1.000
	Week 4	30.41	0.811
	Week 8	11.41	0.987

**Note:** One-way ANOVA, Tukey HSD Post Hoc.

**Table 4:** Findings of the SEG weight values between weeks.

(I) Week	(J) Week	Mean Difference (I-J)	*p*-value
Week 1	Week 4	0.50	1.000
	Week 8	-10.50	0.992
	Week 12	-15.75	0.974
Week 4	Week 1	-0.50	1.000
	Week 8	-11.00	0.991
	Week 12	-16.25	0.971
Week 8	Week 1	10.50	0.992
	Week 4	11.00	0.991
	Week 12	-5.25	0.999
Week 12	Week 1	15.75	0.974
	Week 4	16.25	0.971
	Week 8	5.25	0.999

**Note:** One-way ANOVA, Tukey HSD Post Hoc.

**Table 5:** Results of SE+LCG weight values between weeks.

(I) Week	(J) Week	Mean Difference (I-J)	*p*-value
Week 1	Week 4	-32.25	0.929
	Week 8	-30.33	0.940
	Week 12	-3.91	1.000
Week 4	Week 1	32.25	0.929
	Week 8	1.91	1.000
	Week 12	28.33	0.950
Week 8	Week 1	30.33	0.940
	Week 4	-1.91	1.000
	Week 12	26.41	0.959
Week 12	Week 1	3.91	1.000
	Week 4	-28.33	0.950
	Week 8	-26.41	0.959

**Note:** One-way ANOVA, Tukey HSD Post Hoc.

**Table 6:** Results of intergroup statistical analysis of weight values according to weeks.

Week	Groups	Mean (gram)	SD	F	*p*-value
Week 1	DCG	392.00	41.47	0.155	0.925
	LCG	406.00	66.14		
	SEG	407.66	52.86		
	SE+LCG	414.83	72.61		
Week 4	DCG	417.58	55.05	1.25	0.317
	LCG	372.00	36.59		
	SEG	407.16	66.10		
	SE+LCG	447.08	98.24		
Week 8	DCG	424.33	60.71	0.567	0.643
	LCG	391.00	57.51		
	SEG	418.16	66.70		
	SE+LCG	445.16	98.30		
Week 12	DCG	427.41	69.66	0.119	0.948
	LCG	402.41	71.14		
	SEG	423.41	70.33		
	SE+LCG	418.75	96.86		

**Abbreviations:** DCG: diabetic control group, LCG: l-carnitine group, SEG: swimming exercise group, SE+LCG: swimming exercise+l-carnitine group, SD: standard deviation. One-way ANOVA.

**Table 7:** Results of intragroup statistical analysis of glucose values.

Groups	Tests	Mean (mg/dl)	SD	t	*p*-value
DCG	Pre-test	324.50	78.61	-0.898	0.411
	Post-test	355.33	148.71		
LCG	Pre-test	321.66	91.30	0.183	0.862
	Post-test	312.50	138.66		
SEG	Pre-test	258.66	71.80	0.138	0.896
	Post-test	255.00	134.35		
SE+LCG	Pre-test	223.83	14.38	1.038	0.347
	Post-test	200.16	66.25		

**Abbreviations:** DCG: diabetic control group, LCG: l-carnitine group, SEG: swimming exercise group, SE+LCG: swimming exercise+l-carnitine group, SD: standard deviation. Paired Samples T Test.

**Table 8:** Statistical analysis results of glucose values between groups.

Tests	Groups	Mean (mg/dl)	SD	F	*p*-value
Pre-test	DCG	324.50	78.61	2.941	0.058
	LCG	321.66	91.30		
	SEG	258.66	71.80		
	SE+LCG	223.83	14.38		
Post-test	DCG	355.33	148.71	1.722	0.195
	LCG	312.50	138.66		
	SEG	255.00	134.35		
	SE+LCG	200.16	66.25		

**Abbreviations:** DCG: diabetic control group, LCG: l-carnitine group, SEG: swimming exercise group, SE+LCG: swimming exercise+l-carnitine group, SD: standard deviation. One-way ANOVA.

**Table 9:** Results of intragroup statistical analysis of insulin resistance values.

Groups	Tests	Mean (HOMA-IR)	SD	t	*p*-value
DCG	Pre-test	2.39	0.66	1.280	0.257
	Post-test	1.93	0.55		
LCG	Pre-test	2.45	0.68	0.120	0.923
	Post-test	2.41	1.09		
SEG	Pre-test	1.89	0.61	0.687	0.522
	Post-test	1.80	0.85		
SE+LCG	Pre-test	1.70	0.15	1.762	0.138
	Post-test	1.44	0.48		

**Abbreviations:** DCG: diabetic control group, LCG: l-carnitine group, SEG: swimming exercise group, SE+LCG: swimming exercise+l-carnitine group, HOMA-IR: homeostatic assessment of insulin resistance, SD: standard deviation. Paired Samples T Test.

**Table 10:** Results of statistical analysis of insulin resistance values between groups.

Tests	Groups	Mean (HOMA-IR)	SD	F	*p*-value
Pre-test	DCG	2.39	0.66	2.509	0.088
	LCG	2.45	0.68		
	SEG	1.89	0.61		
	SE+LCG	1.70	0.15		
Post-test	DCG	1.93	0.55	1.561	0.230
	LCG	2.41	1.09		
	SEG	1.80	0.85		
	SE+LCG	1.44	0.48		

**Abbreviations:** DCG: diabetic control group, LCG: l-carnitine group, SEG: swimming exercise group, SE+LCG: swimming exercise+l-carnitine group, HOMA-IR: homeostatic assessment of insulin resistance, SD: standard deviation. One-way ANOVA.

## Data Availability

All data generated or analyzed during this study are included in this published article.

## LIST OF ABBREVIATIONS

BMI: Body Mass Index
DM: Diabetes Mellitus
DM-2: Type 2 Diabetes
EXE: Exercise
LC: L-Carnitine
STZ: Streptozocin
i.p.: Intraperitoneally
DCG: Diabetic Control Group
LCG: L-Carnitine Group
SEG: Swimming Exercise Group
SE+LCG: Swimming Exercise + L-Carnitine Group
SD: Standard Deviation
Sig.: Significance
